# Primary leiomyosarcoma of the portal vein: A progressively enhancing fusiform mass without biliary dilatation as a rare differential for hilar tumors

**DOI:** 10.1016/j.radcr.2026.03.028

**Published:** 2026-04-11

**Authors:** Azumi Miyazawa, Junya Tsuzaki, Ryo Ogawa, Masashi Tamura, Nana Osaki, Keiichi Miyashita, Yuta Abe, Yutaka Nakano, Kensuke Hara, Hidenori Ojima, Shigeo Okuda, Shigeki Sekine, Yuko Kitagawa, Masahiro Jinzaki

**Affiliations:** a Department of Diagnostic Radiology, NHO Tokyo Medical Center, Tokyo, Japan; b Department of Radiology, Keio University School of Medicine, Tokyo, Japan; c Department of Surgery, Keio University School of Medicine, Tokyo, Japan; d Department of Pathology, Keio University School of Medicine, Tokyo, Japan; e Division of Molecular Pathology, Tochigi Cancer Center Research Institute, Tochigi, Japan

**Keywords:** Leiomyosarcoma, Portal vein, Cavernous transformation, Portal hypertension

## Abstract

Primary leiomyosarcomas of the portal vein are exceedingly rare vascular neoplasms. Given the limited number of reports on their imaging features, achieving an accurate preoperative diagnosis is challenging yet essential for determining the optimal treatment strategy. Here, we report a case of an incidentally discovered primary portal vein leiomyosarcoma in an asymptomatic man in his forties. We emphasize the diagnostic significance of its fusiform intraportal morphology. Unenhanced computed tomography (CT) revealed a distinct 45-mm fusiform mass in the hepatic hilum, while dynamic contrast-enhanced CT showed heterogeneous progressive enhancement without biliary dilatation. Magnetic resonance imaging depicted the tumor as hypointense on T1-weighted images and heterogeneously hypointense on T2-weighted images, with notable diffusion restriction. A percutaneous CT-guided biopsy indicated sarcoma with smooth muscle differentiation, and subsequent surgical resection confirmed leiomyosarcoma originating from the tunica media/adventitia of the portal vein. Recognition of a fusiform, progressively enhancing intraportal mass without biliary dilatation can aid in an early and precise preoperative diagnosis.

## Introduction

Leiomyosarcoma is a rare malignant tumor that originates from smooth muscle cells and is predominantly located in the uterus, with less frequent occurrences in the retroperitoneum and thigh [1]. Approximately 2% of leiomyosarcomas develop from the smooth muscle layer of blood vessel walls, known as vascular leiomyosarcomas [2]. The inferior vena cava (IVC) is the primary site of origin for vascular leiomyosarcomas, extensively documented in surgical and radiological literature [3].

In contrast, primary leiomyosarcoma of the portal vein is extremely rare. Owing to the scarcity of documented cases, its usual clinical presentation and distinctive radiological features are largely undefined. This absence of established imaging traits often complicates distinguishing this tumor from bland thrombus or tumor thrombus linked to other malignancies, such as hepatocellular carcinoma (HCC) or intrahepatic cholangiocarcinoma (ICC) [4].

We report a case of portal vein leiomyosarcoma with comprehensive multimodality imaging and pathological correlation. We highlight essential diagnostic characteristics to distinguish this tumor from other masses in the hepatic hilum.

## Case presentation

A man in his forties was referred to our hospital after an incidental discovery of a liver mass during a routine health check-up. The patient's past medical history was remarkable only for childhood appendicitis. Routine laboratory tests revealed no abnormalities.

**Fig. 1:**
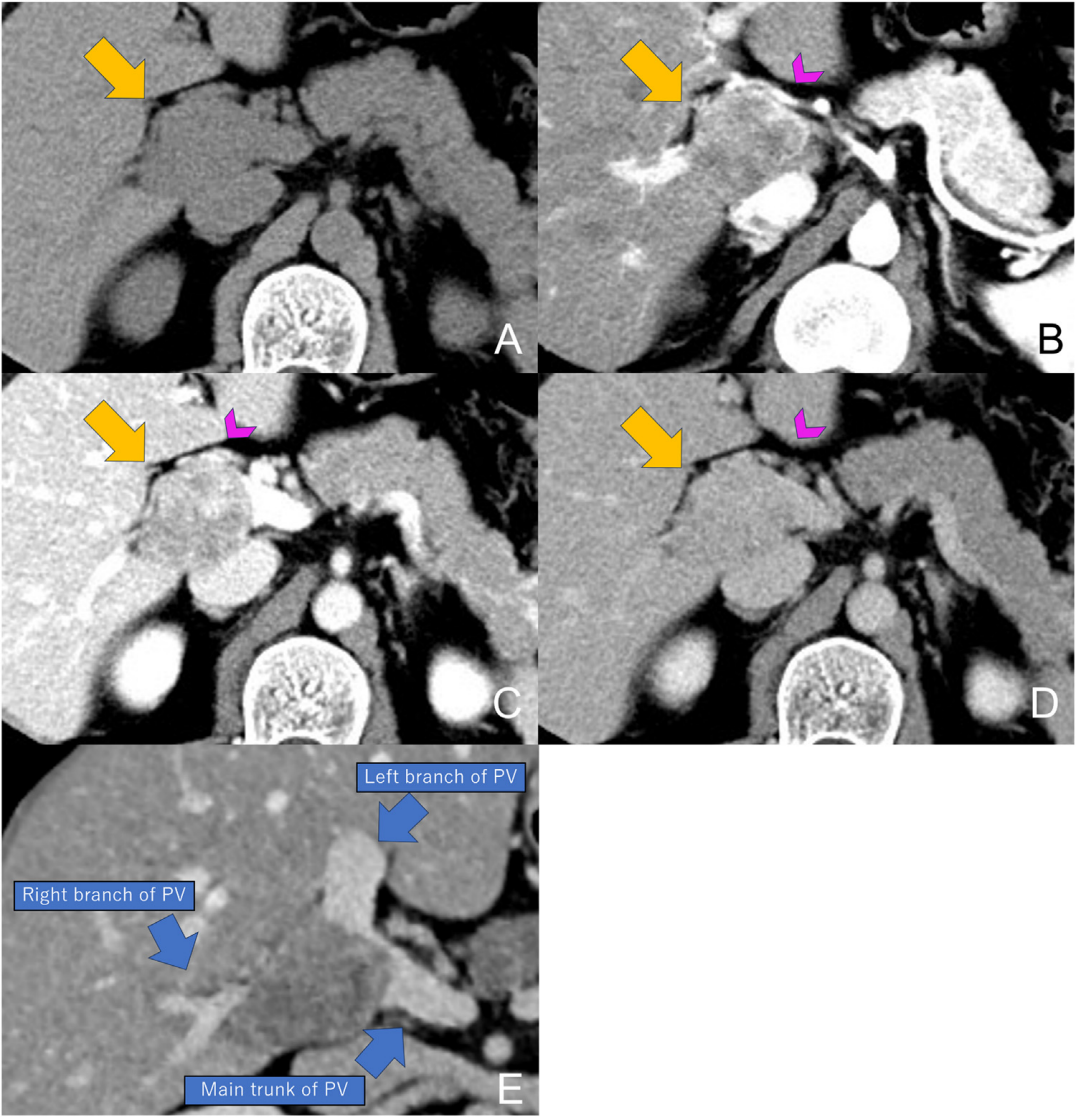
Dynamic CE-CT findings. (A) Unenhanced, (B) arterial, (C) portal venous, and (D) delayed phase images. Unenhanced CT demonstrates a 45-mm low-attenuation mass at the hepatic hilum (yellow arrow). The lesion exhibits heterogeneous and progressively increasing enhancement. Cavernous transformation of the portal vein at the hepatic hilum is also observed (pink arrowheads). The reconstructed view (E) clearly demonstrates the fusiform intraluminal configuration of the tumor and precisely depicts its anatomical relationship to the main portal trunk and the right and left portal branches (blue arrows). PV: portal vein.

**Fig. 2:**
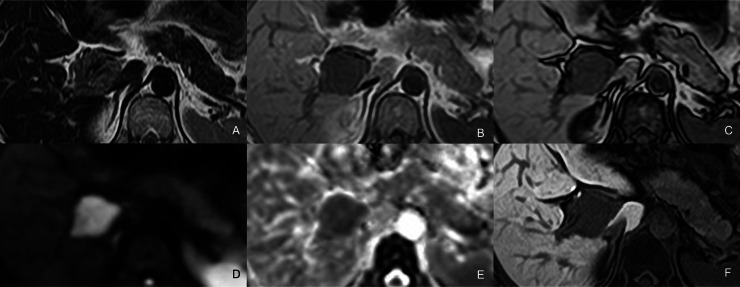
MRI findings. (A) T2-weighted image (T2WI), (B) in-phase and (C) out-of-phase T1-weighted images (T1WI), (D) diffusion-weighted image (DWI), (E) apparent diffusion coefficient (ADC) map, and (F) hepatobiliary phase of gadoxetic acid–enhanced MRI. The mass demonstrates well-defined margins and a fusiform configuration. It appears heterogeneously hypointense on T2WI and homogeneously hypointense on T1WI, with no apparent fat component. The lesion exhibits restricted diffusion. In the hepatobiliary phase (F), the tumor appears hypointense.

Dynamic contrast-enhanced computed tomography (CT) was performed. An intravenous iodinated contrast agent (350 mgI/mL) was administered at a total dose of 600 mgI/kg over 30 seconds. The arterial phase was acquired 15 seconds after the attenuation within a region of interest (ROI) placed in the abdominal aorta at the level of the celiac artery reached the trigger threshold of 160 Hounsfield units. The portal venous phase was obtained 27 seconds after completion of the arterial phase, and the delayed phase was acquired 180 seconds after initiation of contrast injection. The CT revealed a distinct 45-mm mass with heterogeneous enhancement at the porta hepatis (Fig. 1). Cavernous transformation of the portal vein was noted. Subsequently, gadoxetic acid-enhanced magnetic resonance imaging (MRI) was performed. The protocol included fast spin-echo T2-weighted imaging (T2WI), T1-weighted imaging (T1WI) using the Dixon method, and diffusion-weighted imaging (DWI) with a b value of 800 s/mm². No MR angiographic sequences were obtained. The tumor was hypointense on T1WI and heterogeneously hypointense on T2WI, with restricted diffusion (apparent diffusion coefficient value, 1.05 × 10⁻³ mm²/s). No intralesional fat or hemorrhage was identified. The mass showed no uptake in the hepatobiliary phase, which was acquired 15 minutes after gadoxetic acid administration (Fig. 2). It led to occlusion of the right portal vein branch and stenosis of the left branch and main trunk, with collateral vessels developing in the porta hepatis. No intrahepatic bile duct dilatation, liver parenchyma invasion, or IVC involvement was observed. ^18^F-fluorodeoxyglucose positron emission tomography/computed tomography (FDG-PET/CT) indicated increased uptake, with a maximum standardized uptake value (SUVmax) of 6.1. No lymph node or distant metastases were evident on any imaging. Collectively, the imaging findings strongly suggested an intraluminal tumor arising from the portal vein, with leiomyosarcoma as the primary differential diagnosis.

However, given the atypical presentation of the lesion, a preoperative histological diagnosis was deemed necessary to determine the most appropriate surgical procedure and to plan the optimal extent of resection carefully. To ensure maximum safety during the biopsy of this vascular/hilar lesion, the procedure was performed under CT guidance. Careful trajectory planning was conducted by reviewing the intraprocedural CT images to strictly avoid traversing major adjacent blood vessels and the biliary tract. An 18-gauge biopsy needle was utilized, and one core sample was successfully obtained. There were no immediate post-procedural complications. Pathological analysis of the specimen indicated sarcoma with smooth muscle differentiation. Based on this preoperative diagnosis, the patient subsequently underwent resection of the right hepatic and caudate lobes, combined with portal vein resection and reconstruction, extrahepatic bile duct resection, and choledochojejunostomy. Intraoperative findings confirmed that the mass was confined to the portal vein, with no evidence of extension into the surrounding tissues.

**Fig. 3:**
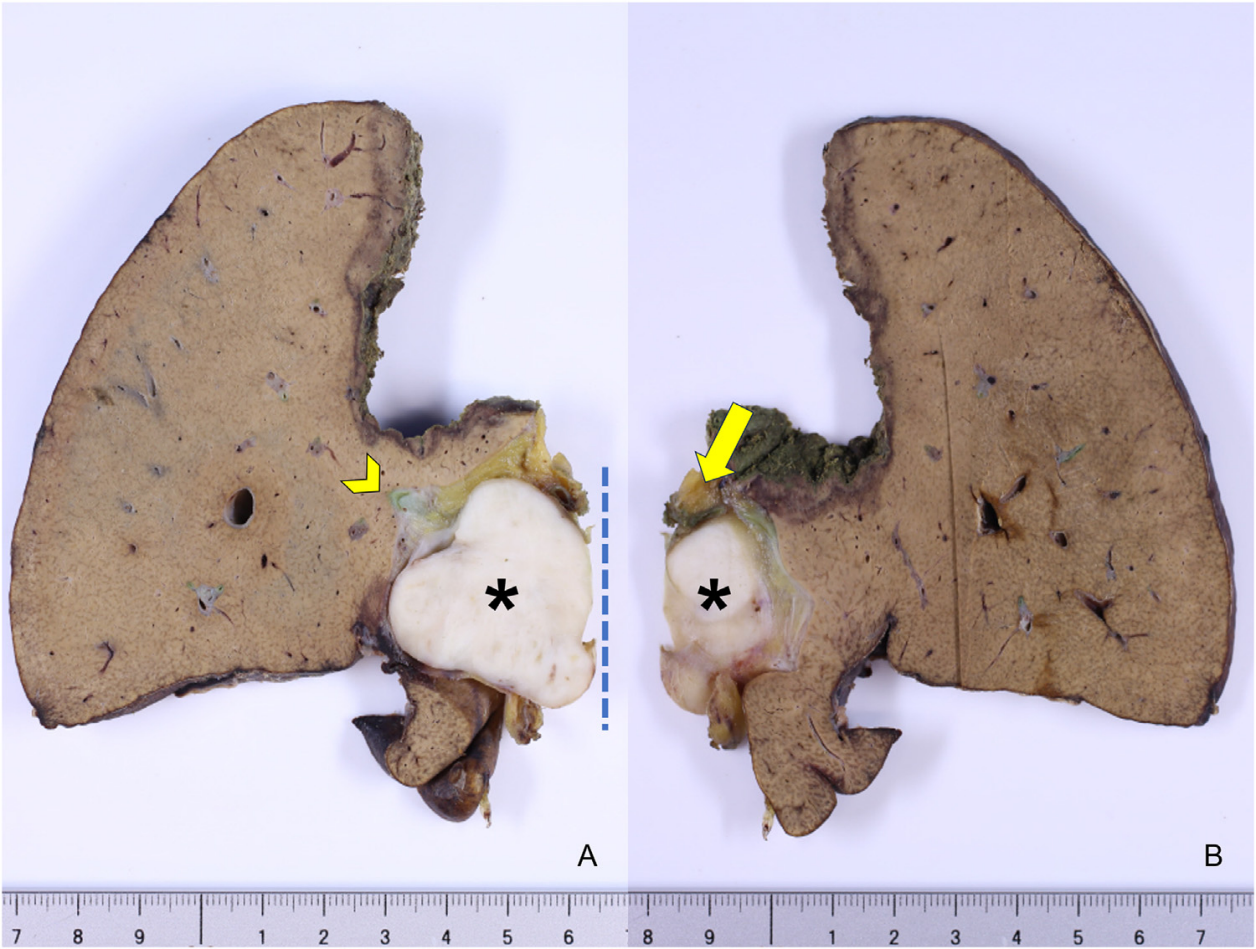
Macroscopic findings. A whitish mass (asterisk) occupying the lumen of the right portal vein, arising from the main portal vein trunk (A), compressing and flattening the extrahepatic bile duct (arrow) without macroscopic evidence of direct invasion (B). The arrowhead indicates the right hepatic duct.

**Fig. 4:**
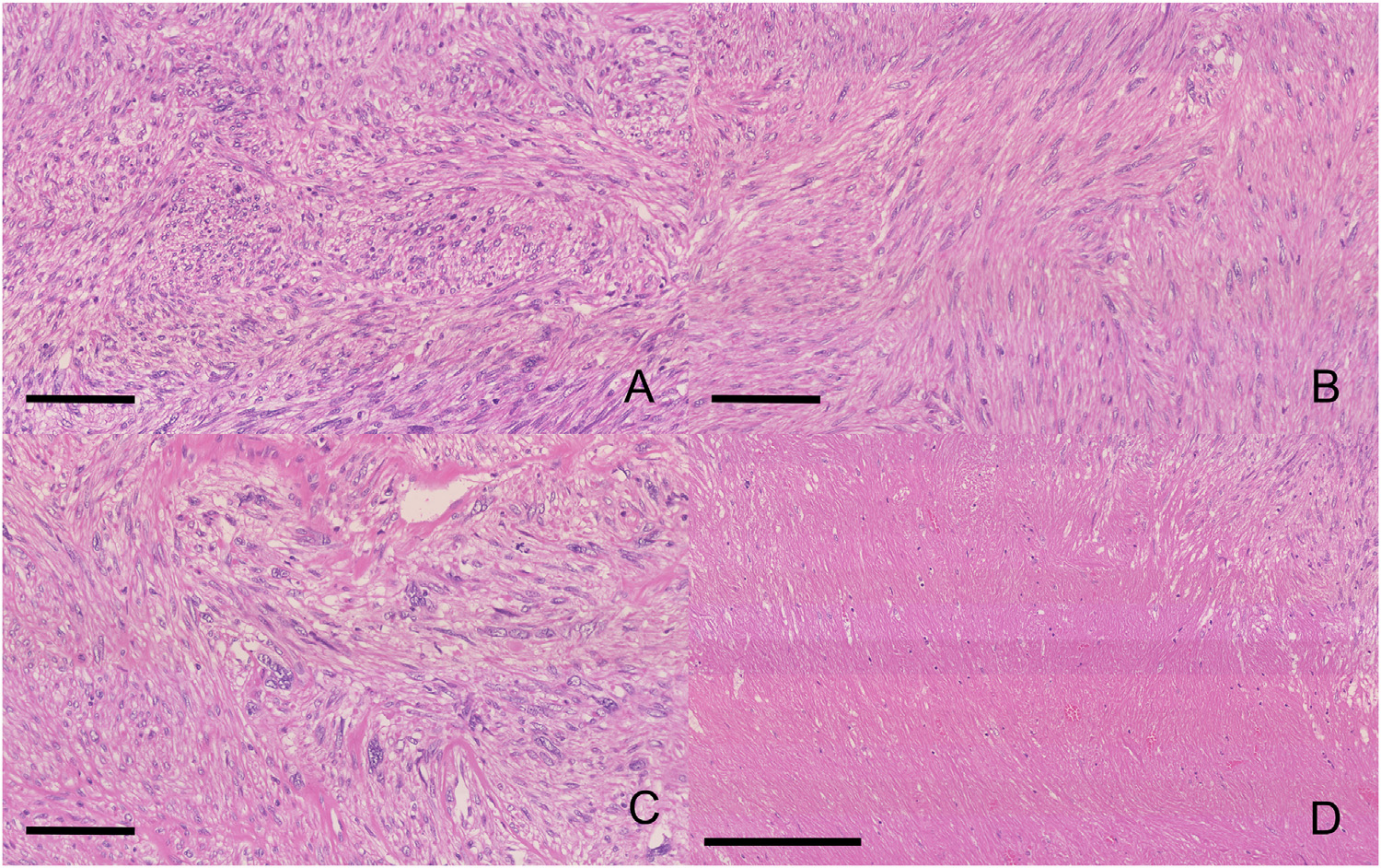
Hematoxylin and eosin staining findings. The tumor is composed of intersecting fascicles of spindle-shaped cells with brightly eosinophilic cytoplasm (A). The tumor shows marked heterogeneity, containing areas with only mild nuclear atypia (B) and regions with severe atypia (C). Focal necrosis is also observed (D). Scale bars: 100 µm (A, B, C), 250 µm (D).

**Fig. 5:**
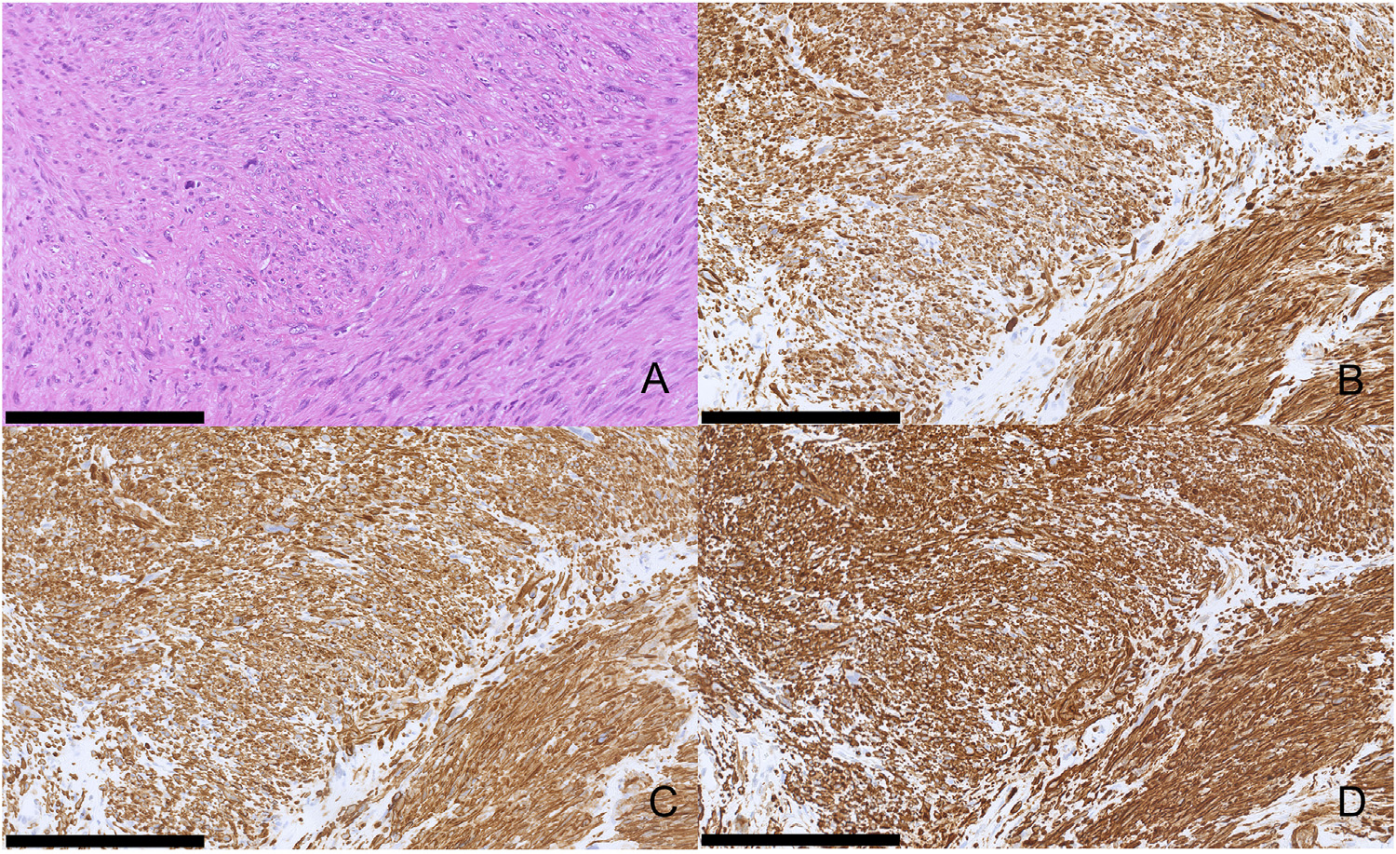
Histological and immunohistochemical findings. (A) Hematoxylin and eosin staining. (B) Desmin staining. (C) α-smooth muscle actin (α-SMA) staining. (D) h-caldesmon staining. Scale bars: 250 µm (A, B, C, D). The tumor cells stain positive for all markers, supporting smooth muscle differentiation.

**Fig. 6:**
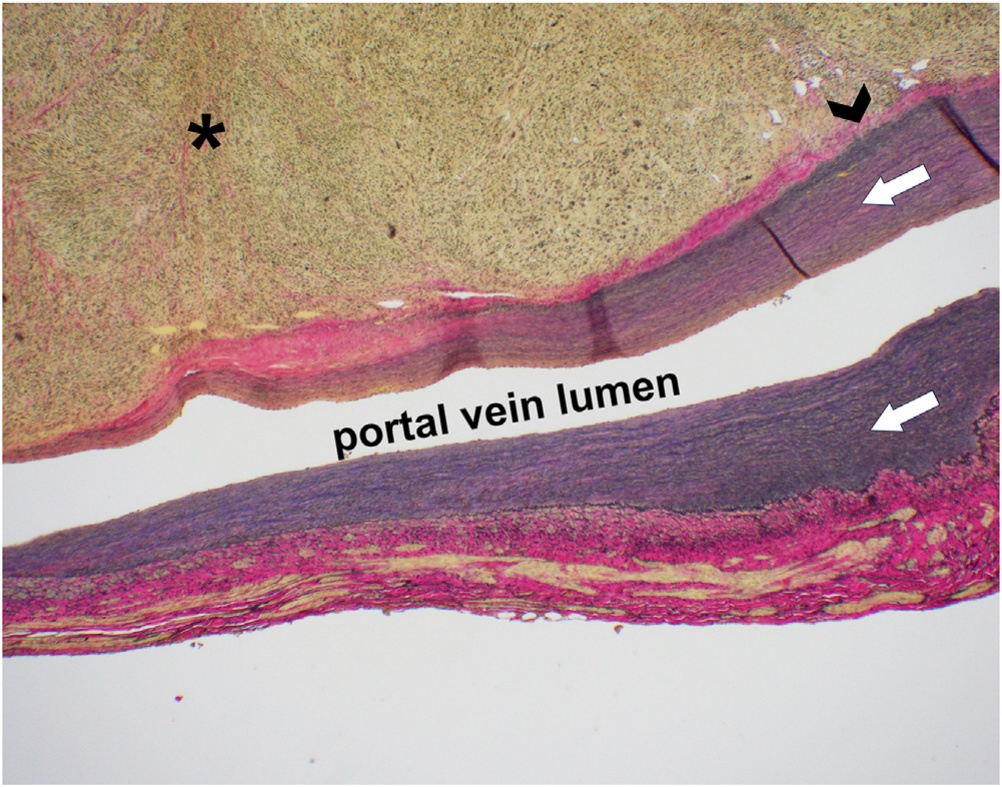
EVG staining. The tumor (asterisk) arises within the tunica media (arrowhead) of the portal vein and exhibits an expansile growth pattern that distends the vessel wall. The arrows indicate the tunica intima.

The resected specimen revealed a distinct whitish tumor mass confined to the portal vein lumen without bile duct wall invasion (Fig. 3). Microscopically, spindle-shaped cells proliferated within the portal vein wall, forming intersecting fascicles with a bright eosinophilic cytoplasm (Fig. 4). Histopathological examination revealed heterogeneous atypia, comprising areas of mild atypia mixed with regions of severe nuclear atypia including bizarre nuclei. The mitotic rate varied regionally, reaching up to 10–12 per 10 high-power fields (HPFs) in the most active areas. Additionally, focal areas of coagulative necrosis were scattered throughout the lesion. Immunohistochemically, the tumor cells tested positive for desmin, α-smooth muscle actin (α-SMA), and h-caldesmon (Fig. 5), confirming the smooth muscle lineage. The Ki-67 labeling index was elevated, reaching 40% in hot spots. Furthermore, Elastica van Gieson staining indicated the tumor originated in the tunica media/adventitia of the portal vein (Fig. 6). Regarding the margin status, tumor components within the portal vein wall extended to the surgical resection margin, indicating a microscopically positive margin (R1 resection). Although a formal risk stratification system was not specifically applied to this case, the combination of a high mitotic rate, tumor necrosis, and significant nuclear atypia is consistent with a high-grade malignancy. Ultimately, the pathology report confirmed the diagnosis as primary leiomyosarcoma of the portal vein. Additionally, a small intrahepatic metastasis histologically similar to that of the primary lesion was incidentally identified in the resected liver.

The patient was monitored without additional treatment. Nineteen months after surgery, CT and MRI scans revealed intrahepatic recurrence, leading to a partial hepatectomy. A second intrahepatic recurrence was detected 32 months after the initial surgery and managed with partial resection.

## Discussion

Primary leiomyosarcoma of the portal vein is extremely rare; to our knowledge, only 10 cases, including this one, have been documented in the literature [[4], [5], [6], [7], [8], [9], [10], [11], [12]] (Table 1). Reported cases of primary portal vein leiomyosarcoma show a male-to-female ratio of approximately 1:2. More than half of the patients were asymptomatic and incidentally diagnosed, while the remainder presented with jaundice or upper abdominal pain. Imataki et al. [11] categorized tumor growth patterns into three types: intraportal, extraportal, and combined. Five intraportal cases, including this one, have been reported [4,5,9,12]. Imaging features of intraportal tumors often include delayed enhancement and cavernous transformation due to portal vein stenosis or occlusion [4,9,12].

**Table 1:** Summary of reported cases of primary portal vein leiomyosarcoma.

Case	Study	Age/ Sex	Location	Symptoms	Size (cm)	Growth pattern	Treatment	Outcome
1	Wilson [5]	28/F	From the portal confluence to the right and left intrahepatic portal branches	Asymptomatic	Long-axis 3	Intraportal	Unresectable	ND
2	Boudjema [6]	44/F	Porta hepatis	Jaundice	Long-axis 3	Extraportal	PD with PV resection (CR)	DOD at 47 mo (Local recurrence at 27 mo, CRT)
3	Gohrbandt [7]	71/F	Porta hepatis	Upper abdominal pain	ND	Combined	Right hepatectomy, PV and bile duct resection (CR)	AWD at 36 mo (Local recurrence at 36 mo, RT)
4	Chiu [4]	67/M	Pancreas head	Asymptomatic	5.3 × 5.9 × 6.4	Intraportal	PD with PV resection	NED at 4 mo
5	Gaignard [8]	53/M	Porta hepatis	Jaundice/Upper abdominal pain	Long-axis 10	ND	Right hepatectomy	NED at 6 mo
6	Esposito [9]	78/M	Liver	Asymptomatic	Long-axis 5.7	Intraportal	Left hepatectomy (R0)	NED at 18 mo
7	Tzedakis [10]	42/F	Liver	Jaundice	Long-axis 3	ND	Right hepatectomy, PV resection (R0)	NED at 20 mo
8	Imataki [11]	59/F	Pancreas head	Asymptomatic (elevated serum amylase)	3.5 × 2.9	Combined	PD with PV resection	AWD at 46 mo (Local recurrence at 38 mo, RT)
9	Adnor [12]	56/F	Porta hepatis	Abdominal pain/Right upper quadrant mass	ND	Intraportal	Chemotherapy	ND
10	Our case	40/M	Porta hepatis	Asymptomatic	4 × 4 × 4	Intraportal	Right hepatectomy, caudate lobectomy, PV and extrahepatic bile duct resection	AWD at 32 mo (Intrahepatic recurrence at 19, 32 mo, Re-resection)

AWD, alive with disease; CR, complete resection; CRT, chemoradiotherapy; DOD, dead of disease; F, female;

M, male; ND, not described; NED, no evidence of disease; PD, Pancreaticoduodenectomy; PV, portal vein; R0, microscopically margin-negative resection; RT, radiotherapy; mo, months.

Based on this classification, the current case was categorized as the intraportal type. CT and MRI scans revealed cavernous transformation of the portal vein, the absence of biliary duct dilatation, and a mass with heterogeneous enhancement and prolonged enhancement in the delayed phases. The fusiform shape of the tumor correlated with the pathological findings, indicating its origin within the tunica media/adventitia of the portal vein and its expansive growth within this layer (Fig. 6). This growth pattern implied that the tumor derived from smooth muscle cells located in the media of the portal vein.

When evaluating a mass involving the portal venous system, a precise differential diagnosis is essential. HCC, ICC, and metastatic liver tumors are key considerations for intrahepatic lesions. In establishing the preoperative diagnosis, it was crucial to differentiate the lesion from a bland thrombus and a tumor thrombus originating from hepatocellular carcinoma (HCC) or intrahepatic cholangiocarcinoma (ICC). First, a bland portal vein thrombus was considered highly unlikely due to the lesion's enhancement behavior and expansile intraluminal growth; bland thrombi typically do not exhibit internal contrast enhancement and rarely expand the vessel to such an extent. Second, a tumor thrombus secondary to a primary hepatic malignancy was confidently ruled out because there was a complete absence of a concurrent hepatic parenchymal mass. Furthermore, the lack of associated biliary dilatation strongly argued against an advanced ICC. For masses within the porta hepatis, potential differentials include schwannomas from the hepatoduodenal ligament, Castleman disease, and neuroendocrine neoplasms (NENs) of biliary origin. Neurogenic tumors typically exhibit heterogeneous high-signal intensity on T2WI. In contrast, the present case demonstrated heterogeneous hypointensity on T2WI, which may reflect intratumoral heterogeneity, including fibrosis and necrosis. Castleman disease commonly manifests as a homogeneously hypervascular mass < 5 cm, while larger lesions often show heterogeneous enhancement with necrosis [13]. Notably, the absence of calcifications, frequently seen in Castleman disease, was observed in this case. NENs typically present with jaundice, upstream biliary dilatation, and commonly originate from the common bile duct [14], which is inconsistent with the patient’s clinical presentation. Collectively, these specific imaging features strongly supported our preoperative diagnosis of a primary portal vein leiomyosarcoma.

To further evaluate the physiological impact of the portal vein occlusion and the observed cavernous transformation, we carefully assessed for signs of portal hypertension. Retrospective review of the abdominal CT revealed no evidence of splenomegaly, portosystemic varices, or ascites. Furthermore, the patient's platelet count was within the normal range (17.7 × 10⁴/µL). Collectively, these objective findings indicate that despite the presence of cavernous transformation, the patient did not exhibit systemic portal-hypertension physiology. This suggests that the developed collateral vessels sufficiently compensated for the chronic portal venous obstruction, maintaining adequate portal hemodynamics.

A review of the literature suggests that complete surgical resection represents the sole curative option [6,7,9,10]. Chemotherapy or radiotherapy has been utilized in select instances [6,7,11]. Owing to the scarcity of evidence regarding the management of recurrent portal vein leiomyosarcoma, treatment approaches are predominantly inferred from leiomyosarcomas originating in other venous structures. In IVC leiomyosarcoma, long-term survival has been achieved through repeat surgery [15]. Early detection is essential for aggressive interventions. Similarly, for IVC leiomyosarcoma, strict follow-up with FDG-PET/CT has been reported to be effective in identifying recurrent lesions at an early stage, contributing to improved survival outcomes [16]. Furthermore, a case involving primary hepatic leiomyosarcoma treated with transcatheter arterial chemoembolization has been documented [17].

Although the presence of synchronous intrahepatic metastasis at the time of the initial resection indicated advanced disease, formal staging was not specifically applied due to the extreme rarity of this primary tumor. Despite the high-grade histological features and the microscopically positive margin (R1), adjuvant therapy was not administered because there is currently no established evidence or standard chemotherapy regimen for this rare entity. Instead, a strategy of close postoperative surveillance was adopted. The follow-up protocol consisted of contrast-enhanced computed tomography (CT) performed every 4 to 6 months. When new intrahepatic metastases were suspected on surveillance CT, gadoxetic acid-enhanced magnetic resonance imaging (EOB-MRI) was additionally performed for precise evaluation and early detection.

Given the limited number of reported cases, further investigations are necessary to refine the imaging spectrum, identify prognostic factors, and establish optimal treatment modalities for this rare condition.

## Conclusion

Primary leiomyosarcomas of the portal vein are uncommon. Important imaging characteristics, such as fusiform morphology, heterogeneous progressive enhancement, and cavernous transformation without biliary dilatation, offer crucial diagnostic clues. Identifying these features can aid radiologists in distinguishing this uncommon condition from other tumors in the hilum and in expediting surgical consultation and treatment.

## Patient consent

Complete written informed consent was obtained from the patient for the publication of this study and accompanying images.
